# Optimal energy delivery and measured energy expenditure—impact of length of stay

**DOI:** 10.1186/s13054-017-1612-6

**Published:** 2017-02-22

**Authors:** Mette M. Berger, Claude Pichard, Eric Fontaine

**Affiliations:** 1 Service of Adult Intensive Care, Lausanne University Hospital, Lausanne, Switzerland; 2 Nutrition Unit; Geneva University Hospital, Geneva, Switzerland; 3 Unité de Nutrition Artificielle, Grenoble University Hospital, and INSERM 1055 Univ. Grenoble Alpes, Grenoble, France; 4 Service of intensive care medicine, Lausanne University Hospital (CHUV), Rue du Bugnon 46, 1011 Lausanne, Switzerland

The issue of optimal energy delivery in critical care patients is a matter of debate, and guidelines recommend to base energy prescriptions on measured energy expenditure (EE). Recently, the largest study ever (*n* = 1171 patients) of the relation between energy and protein delivery, measured EE, and outcome was published [1]. The authors should be commended for this contribution, particularly for confirming the importance of proteins to outcome. Nevertheless, although they tried “to reduce any possible bias caused by short stay” by including in the analysis only patients staying >96 h, the interpretation of their results was probably contaminated by short stayers, as the median reported length of stay was 5 days.

The authors calculated the percentage of administered calories by resting EE (%ADCal/REE): each patient was assigned one value representing the mean of the stay’s delivered kcal. They report a U-shaped curve of mortality by %AdCal/REE, the lowest mortality being observed for 70% of the measured EE value. Despite considering only patients staying >96 h, and a very efficient feeding protocol (progression to target within 4 days), this mathematically induces a bias as shown in Fig. 1. This would occur despite their rapid progression to target (much faster than in most studies); the daily mean (DM) would be close to 88% by day 11. With a median stay of 5 days, the daily mean intake would be about 74% of target: these less severe patients are discharged because they do not require ICU treatment and not because they receive 85% of target. This is typically what was observed in trials based on equations showing that “less is more”: Krishnan et al. [2] showed that a moderate caloric intake (i.e., 33 to 65% of the American College of Chest Physicians (ACCP) targets; ≅9 to 18 kcal/kg/day) was associated with better outcome. Based on similar equation targets, Heyland et al. [3] showed an optimal mortality around 85% of target. These data do not fit though with the Swiss supplemental parenteral nutrition study [4], which showed that feeding to measured target after day 3 versus feeding about 80% of target in control was associated with a significant reduction of infectious complications (both groups starting with a −4000 kcal cumulated deficit).

**Fig. 1:**
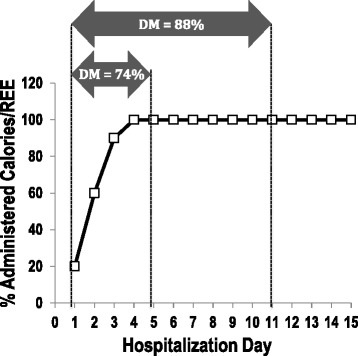
Example of calories administered to a patient. The daily mean (*DM*) of calories is 74% if the patient leaves the ICU at day 5 but 88% if the patient leaves at day 11. The only way to make the mean unaffected by the length of stay is to calculate the mean only once the delivered calories have reached a plateau

Our suggestion would be to redo the outcome analysis while including only in their regression the “mean kcal value of stay” of the stable feeding days, and not feed progression days. Possibly the results would show the lowest mortality somewhere between 95 and 105% of measured EE.

## Abbreviations

%AdCal/REE: Administered calories divided by resting energy expenditure
DM: Daily mean
EE: Energy expenditure
